# The Predictive Value of SPECT/CT imaging in colorectal liver metastases response after ^90^Y-radioembolization

**DOI:** 10.1371/journal.pone.0200488

**Published:** 2018-07-10

**Authors:** Piotr Piasecki, Jerzy Narloch, Krzysztof Brzozowski, Piotr Zięcina, Andrzej Mazurek, Anna Budzyńska, Jan Korniluk, Mirosław Dziuk

**Affiliations:** 1 Interventional Radiology Department of Military Institute of Medicine, Warsaw, Poland; 2 Nuclear Medicine Department of Military Institute of Medicine, Warsaw, Poland; 3 Oncology Department of Military Institute of Medicine, Warsaw, Poland; North Shore Long Island Jewish Health System, UNITED STATES

## Abstract

The aim of this study was to evaluate a modified method of calculating the ^99m^Tc/^90^Y tumor-to-normal-liver uptake ratio (mT/N) based on SPECT/CT imaging, for use in predicting the overall response of colorectal liver tumors after radioembolization. A modified phantom-based method of tumor-to-normal-liver ratio calculation was proposed and assessed. In contrast to the traditional method based on data gathered from the whole tumor, gamma counts are collected only from a 2D region of interest delineated in the SPECT/CT section with the longest tumor diameter (as specified in RECIST 1.1). The modified tumor-to-normal-liver ratio (mT/N1) and ^90^Y predicted tumor absorbed dose (PAD) were obtained based on ^99m^Tc-MAA SPECT/CT, and similarly the modified tumor-to-normal-liver ratio (mT/N2) and ^90^Y actual tumor absorbed dose (AAD) were calculated after ^90^Y-SPECT/CT. Tumor response was assessed on follow-up CTs. Using the newly proposed method, a total of 103 liver colorectal metastases in 21 patients who underwent radioembolization (between June 2009 and October 2015) were evaluated in pre-treatment CT scans and ^99m^Tc-MAA-SPECT/CT scans and compared with post-treatment ^90^Y-SPECT/CT scans and follow-up CT scans. The results showed that the mT/N1 ratio (p = 0.012), PAD (p < 0.001) and AAD (p < 0.001) were predictors of tumor response after radioembolization. The time to progression was significantly lengthened for tumors with mT/N1 higher than 1.7 or PAD higher than 70 Gy. The risk of progression for tumors with mT/N1 lower than 1.7 or PAD below 70 Gy was significantly higher. The mT/N2 ratio had no significant correlation with treatment results.

**Conclusion:**

The mT/N1 ratio, PAD, and AAD can be used as predictors of tumor response to SIRT treatment, and SPECT/CT imaging can be used for dosimetric assessment of radioembolization.

## Introduction

The main advantage of Yttrium-90 in palliative treatment of liver tumors is its pure beta-radiation emission with high energy and short half-life[1, 2]. The main limitation of radioembolization is the low tolerance of healthy liver tissue to radiation[3–5]. Therefore, proper calculation of the activity of ^90^Y-microspheres, which can destroy liver tumors while sparing healthy liver tissue, is very important. The absence of primary gamma emission of ^90^Y leads to significant problems not only with post-treatment imaging of therapy effects but also with calculation of dosimetry parameters for liver parenchyma[6–8]. Consequently, tumor dosimetry is not obtained in everyday practice. Dosimetry based on the MIRD equation (Medical Internal Radiation Dosimetry) and SPECT/CT imaging appears to be the best option for most centers performing radioembolization, but to attain wider use, it should be easier to calculate[7, 9, 10].

Patients undergoing radioembolization typically undergo a pre-treatment ^99m^Tc-MAA SPECT/CT scan to calculate the ^90^Y dose. The tumor-to-normal-liver uptake ratio (mT/N1) obtained in this procedure may serve as a basis for calculating further dosimetric parameters, including the predicted tumor absorbed dose (PAD) of ^90^Y. A second SPECT/CT (bremsstrahlung) scan is performed after radioembolization to assess the post-treatment ^90^Y-microsphere distribution. On this basis the tumor-to-normal-liver ratio (mT/N2) and actual adsorbed dose of ^90^Y (AAD) can also be calculated and compared with pre-treatment and follow-up data [1, 7, 9, 10].

With early dosimetric evaluation, patients with high potential risk of radiation-induced liver disease or patients with non-curative tumor doses can be recognized shortly before or immediately after undergoing a selective internal radiation therapy (SIRT) procedure. In view of recent reports on SIRT from prospective trials (SIRFLOX/FOXFIRE), it is important to develop a method for selecting patients who would benefit from the therapy[11, 12]. In this study, we propose and assess a new approach involving SPECT/CT for predicting the effects of radioembolization in patients with liver tumors, based on a simplified method of calculating the tumor-to-normal-liver ratio.

## Materials and methods

The study was approved by Ethical Committee Board of Military Institute of Medicine (decision: 24/WIM/2009). All procedures performed in studies involving human participants were in accordance with the ethical standards of the institutional and o/or national research committee and with the 1964 Helsinki declaration and its later amendments or comparable ethical standards. Informed consent was obtained from all individual participants included in the study.

### Phantom dosimetric study

We first performed a phantom study to determine if gamma counts obtained only from the middle-of-sphere section could be used for dosimetric calculations in ^90^Y radioembolization. The IEC Body Phantom was used, filled with six different volumes of calibrated amounts of ^90^Y resin microspheres. The inner diameters of the fillable spheres were 10 mm, 13 mm, 17 mm, 22 mm, 28 mm and 37 mm. The activity in the spheres was 3.8 MBq/ml, while the background activity was 0.038 MBq/ml. Gamma counts were collected from each phantom section containing spheres, and the sphere-to-background count ratio was calculated (phT/N).

The two smallest spheres of the IEC Phantom were not clearly visible and were excluded from the study.

### Study population

From June 2009 to December 2015 twenty-one patients with unresectable liver metastases of colorectal cancer were enrolled. A total of 103 tumors were selected and evaluated.

### Study protocol

Radioembolization was conducted in line with guidelines approved by a panel of experts and the local ethics committee. One session per patient of resin microsphere (Sirtex, Australia) treatment was used for the study. As a pre-treatment procedure, 120-180 MBq of ^99m^Tc-MAA was administered into the common hepatic artery. In the radioembolization procedure, resin microspheres were injected using the same microcatheter position. SPECT/CT was performed < 25 min after ^99m^Tc/^90^Y administration.

The Body Surface Area Method was used for calculation of ^90^Y activity[9, 10]. As the first step, a region-of-interest (ROI) analysis of the tumor-to-normal-liver ratio was used on the SPECT images to determine the standard ratio (sT/N)—a detailed description is available elsewhere [7,9]. The modified tumor-to-normal-liver uptake ratio (mT/N) was then calculated using a new method proposed by the authors, as described below.

The RECIST 1.1 criteria were used to evaluate target liver lesions. Correlations between dosimetric parameters of target liver lesions detected with ^99m^Tc/^90^Y-SPECT/CT were investigated and compared with initial and follow-up CT results. The target liver lesions were those that had a longest diameter of at least 10 mm and were clearly visible on CT and ^99m^Tc/^90^Y-SPECT/CT. Tumors with areas of necrosis were not excluded (9%)[13].

For SPECT/CT imaging the GE Infinia Hawkeye 4 was used, with 1-inch NaI crystal detectors and a low-dose four-slice CT protocol. The protocols for ^99m^Tc-MAA imaging and ^90^Y bremsstrahlung image acquisition are shown in Table 1.

**Table 1:** Details of ^99m^Tc-MAA imaging and ^90^Y bremsstrahlung.

^99m^ Tc-MAA imaging	^90^Y-SPECT/CT bremmstrahlung
Energy window: 140 keV +/- 15%; collimator type: LEHR; heads in H mode **Whole body imaging (WB)** Scan mode: continuous; exposure time per pixel: 240 sec; speed: 10 cm/min; body contour on **ANT-POST SPOT abdomen imaging** Stop on counts: 800 kcts, reached on each detector independently Scan location: H mode, start angle 0^o^; body contour off; matrix 256x256, zoom 1.0 **LLAT-RLAT SPOT abdomen imaging** Stop on counts: 800 kcts, reached on each detector independently Scan location: H mode, start angle 270^o^; body contour off; matrix 256x256, zoom 1.0 **ANT-POST SPOT chest imaging** Stop on time based on the ANT-POST SPOT abdomen imaging duration Scan location: H mode, start angle 0^o^; body contour off; matrix 256x256, zoom 1.0 **SPECT/CT abdomen imaging** TOMO COR correction on; start angle 0^o^; body contour on Scan mode: step and shoot, 30 sec per projection Total angular range 360^o^, arc per detector 180^o^, view angle 6^o^, numer of views: 60 Matrix 128x128, zoom 1.0 Emission first, followed by CT/AC CT Scan type: axial; slice thickness: 5.0 mm X ray voltage 140 kV, current 2.5 mA; rotation velocity: 2.6 RPM CT reconstruction: matrix 512x512, pixel size 1.10 mm, filter stnd	**TOMO** Collimator type: HEGP, COR correction on Energy window: 140 keV +/- 100% Heads in H mode; start angle 0^o^; body contour on Scan mode: step and shoot, 30 sec per projection Total angular range 360^o^, arc per detector 180^o^, view angle 6^o^, numer of views: 60 Matrix 128x128, zoom 1.0 Emission first, followed by CT/AC **CT** Scan type: axial; slice thickness: 5.0 mm X ray voltage 140 kV, current 2.5 mA; rotation velocity: 2.6 RPM CT reconstruction: matrix 512x512, pixel size 1.10 mm, filter stnd

For image post-processing, a Xeleris 3.0423 Workstation (GE, USA) with Volumetrix MI software was used. The ordered subset expectation maximization (OSEM) method of iterative reconstruction was applied, with 2 iterations and 10 subsets. The prefilter HANN 0.9 and the 3D postfilter BUTTERWORTH 0.48 were used.

### Patient dosimetric study

The main steps in calculating dosimetric parameters were as follows: 1. Selection of target tumors in pre-treatment CT scans and identification of them with ^99m^Tc/^90^Y-SPECT/CT. 2. Calculation of the modified tumor-to-normal-liver ratio (mT/N1 and mT/N2). 3. Calculation of the predicted and actual absorbed dose for the target liver tumors (PAD and AAD, respectively). 4. Evaluation of tumor responses using data obtained in CT scans and of tumor dosimetric parameters [7, 14].

**Fig 1:**
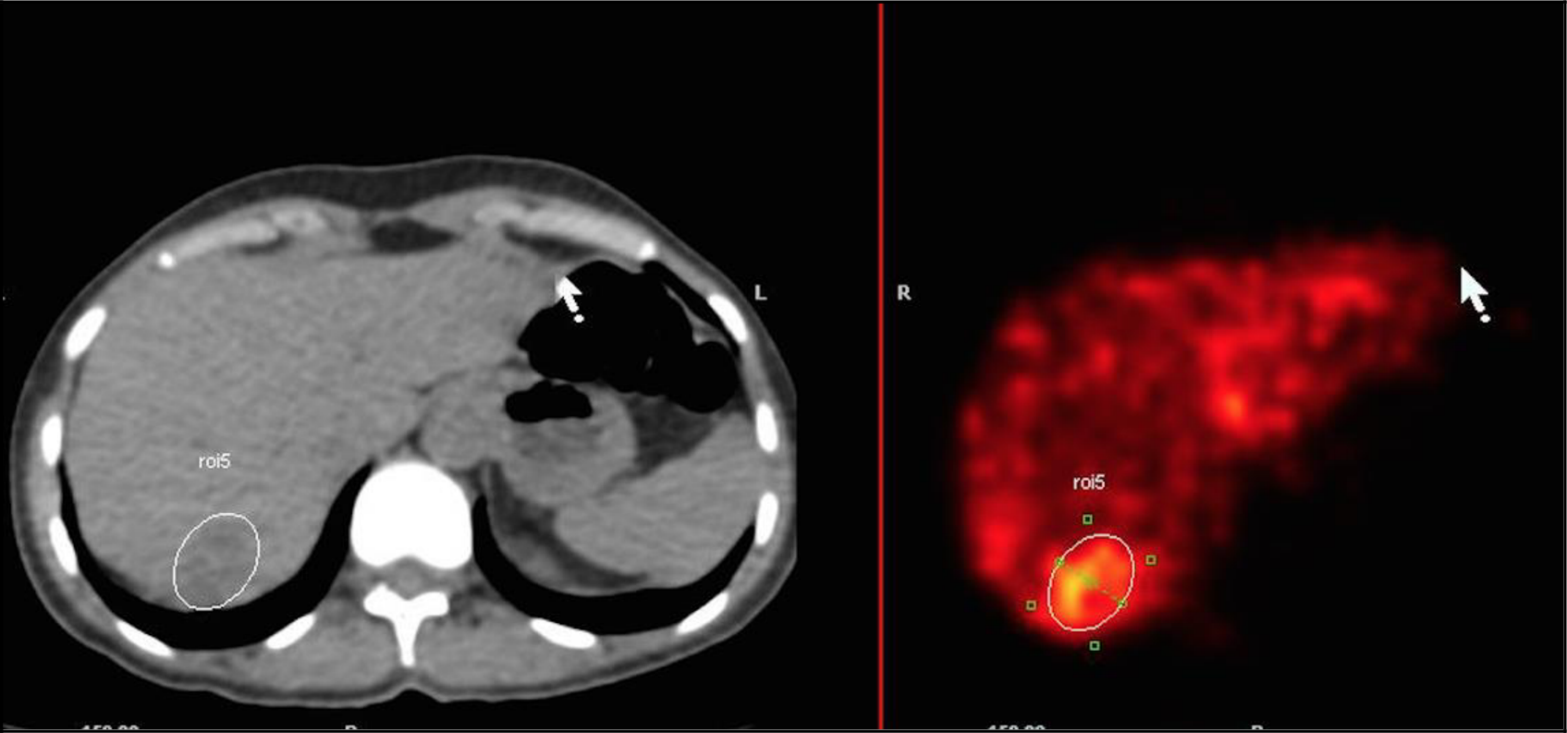
Gamma counts measurement in SPECT/CT after establishing ROI on CT scan with the longest tumor diameter.

To calculate mT/N1, the slice with the longest target tumor diameter was selected in ^99m^Tc-MAA SPECT/CT. Its borders were delineated on CT using semi-automatic tools: a 2D ROI was drawn manually on a CT image generated with an abdominal CT window (Volumetrix MI application on Xeleris 3.0423 Workstation). In the next step, the ROI was automatically duplicated for the fusion ^99m^Tc-MAA scans, and counts of gamma emission from within it were obtained (see Fig 1). Another ROI was placed in an area of healthy liver tissue adjacent to the assessed liver tumor, within the same liver segment, and the gamma emission counts from within this second ROI were determined. The mT/N1 ratio for ^99m^Tc-MAA SPECT/CT was then calculated using the following formula: 1) [7, 14–16].

$$\mathrm{mT}/N1=\frac{counts\;in\;tumor^{\prime }s\;ROI}{counts\;in\;healthy\;liver^{\prime }s\;ROI}$$ (1)

mT/N1 – modified tumor to normal liver uptake ratio in ^99m^Tc-MAA SPECT/CT

To determine the mT/N2 ratio for ^90^Y-SPECT/CT, all the steps listed above were repeated. The liver absorbed dose (LAD1 or LAD2: predicted or actual, respectively), tumor predicted absorbed dose (PAD), and tumor actual absorbed dose (AAD) were calculated according to the following formulas, using the volume as a surrogate for the mass: 2-3) [7, 14–16].

$$\mathrm{LAD}1/\mathrm{LAD}2[Gy]=\frac{{}^{90Y}A[GBq]\times 49,67\times (1-LB)}{V_{Healthy\;liver}+{\left(mT/N\times V_{tumor}\right)}_{n}}$$ (2)

LAD1/LAD2- ^90^Y liver absorbed dose (Gy) calculated separately for ^99m^TC-MAA and ^90^Y-SPECT/CT

^90Y^A – ^90^Y activity to administer (GBq)

LB – lung breakthrough

mT/N – modified-tumor-to-normal uptake ratio (mT/N1 or mT/N2 respectively)

n – number of liver tumors

$${TD}_{PAD/RAD}(Gy)=mT/N\times LAD$$ (3)

TD_PAD/AAD_ - ^90^Y tumor predicted or actual absorbed dose (Gy), respectively

### Statistical analysis

Continuous variables are reported as mean ± SD, and categorical variables as percentages. ANOVAs or Student’s t-test were used for continuous variables if the distributions were suitable; if not, the Kruskal-Wallace test or Mann-Whitney U test were used. For comparisons of categorical variables, the Wilcoxon signed-rank test was used. Median OS (overall survival) and PFS (progression-free survival) were computed using the Kaplan–Meier estimator. The log rank test was used for comparisons of time to progression (TTP_tumor_). Correlations between variables were assessed with Spearman’s correlation coefficient. Multivariate correspondence analysis was used for tumors to assess complete response (CR) and disease progression (PD). Receiver operating characteristic (ROC) curves were generated for PAD and mT/N1. We performed calculations for PAD based on the generally accepted threshold of 70 Gy [6].

P values less than 0.05 were considered significant. Statistical calculations were carried out using STATISTICA 12.0 software.

## Results

### Phantom data

**Fig 2:**
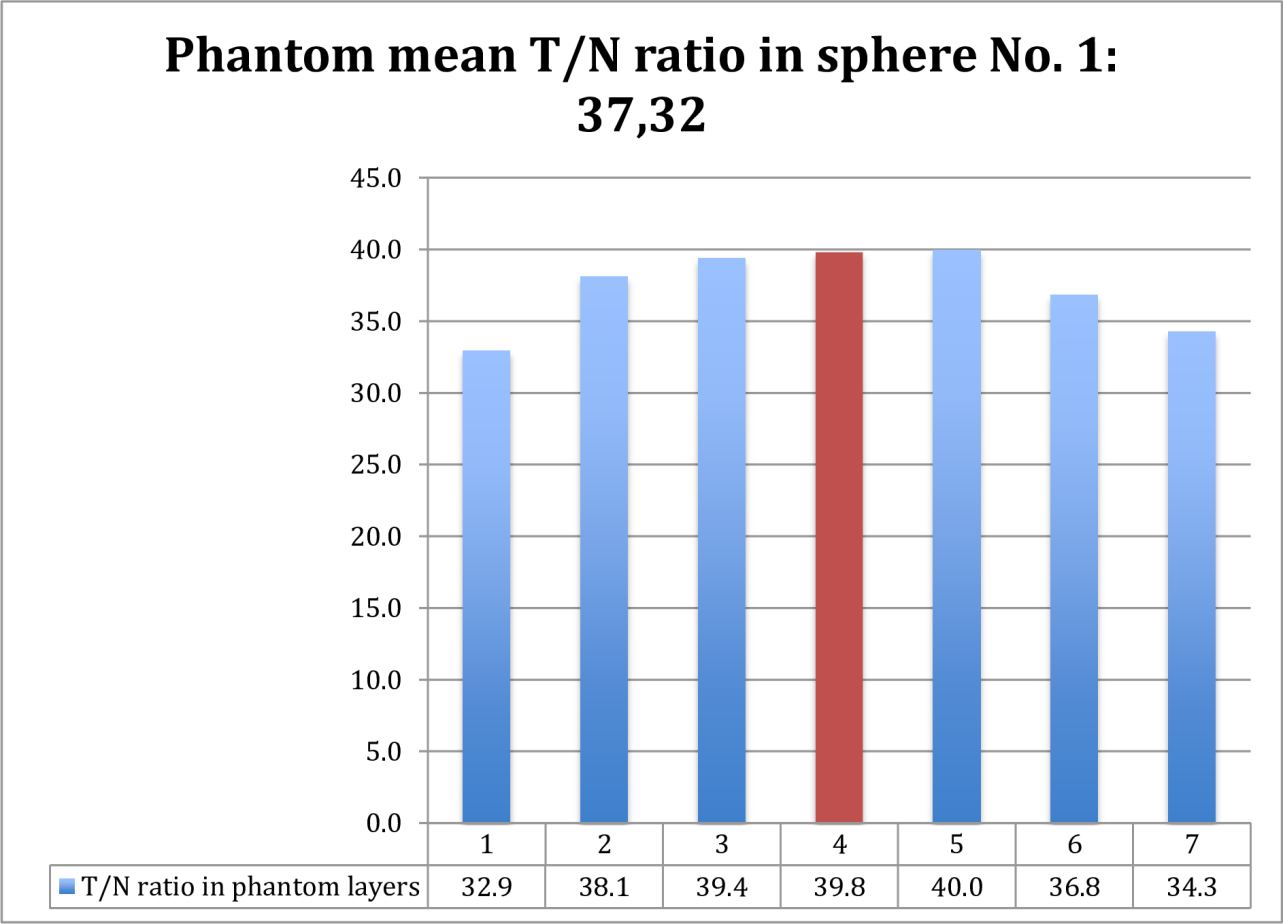
“Tumor-to-normal-tissue-ratio in the phantom: phT/N” in particular layers of sphere No.1 of IEC-phantom. There is no significant difference between T/N ratio of median layer (red) and mean phantom total T/N ratio of sphere No. 1. (p = 0.148, Student t-test).

As shown in Table 2 and Fig 2, gamma counts from the middle (widest diameter) section closely matched those obtained from the entire sphere, indicating that they could be used to calculate the liver tumor absorbed dose.

**Table 2:** Phantom data.

Sphere No.	Volume (ml)	Diameter (mm)	^90^Y Activity (GBq)	phT/N (±SD) total	phT/N median layer	p
1	25.52	37	100.76	37.33 (±2.8)	39.80	0.148
2	11.49	28	43.662	21.11 (±2.01)	22.15	0.261
3	5.58	22	21.204	14.36 (±0.78)	15.10	0.106
4	2.57	17	9.766	9.08 (±0.47)	9.40	0.335

### Patient data

The SIRT procedures were performed with technical success, and no serious adverse events were observed. Overall survival (OS) was 17.3 months and progression-free survival (PFS) was 6 months. The clinical and treatment data are summarized in Table 3.

**Table 3:** Patient clinical and treatment data.

VARIABLE	VALUE	p-value^1^
GENDER (M/F)	17/4	nd
AGE		nd
Mean	56	
Range	35-67	
Median	54	
LIVER VOLUME (ml)		ns^2^
Mean	1712	
Range	920-2424	
Median	1714	
TUMOR VOLUME (ml)		0.03^3^
Mean	276	
Range	7-991	
Median	208	
% OF LIVER INFILTRATION		0.04^3^
Mean	15	
Range	1-52	
Median	12	
INJECTED 90Y ACTIVITY (GBq)		ns^2^
Mean	1.8	
Range	1.0-2.8	
Median	1.8	
PREDICTED LIVER ABSORBED DOSE (Gy)		0.04^3^
Mean	43	
Range	21-64	
Median	48	
ACTUAL LIVER ABSORBED DOSE (Gy)		ns^3^
Mean	44	
Range	21-63	
Median	49	
LUNG SHUNT (%)		ns^3^
Mean	7.4	
Range	3.5-13	
Median	7	
TREATMENT RESULTS		
CR	0	
PR	4	
SD	14	
PD	3	

^1^- correlations between variable and tumor response (according to RECIST1.1)

^2^ – ANOVA^,^

^3^ – U-Mann-Whitney test

### Tumor and dosimetric data

Initially 112 liver tumors within 21 patients were found, but for 9 tumors the target lesion did not meet the inclusion criteria. Thus, data for 103 liver tumors were analyzed. (Table 4)

**Table 4:** Radiological response parameters and the dosimetric calculations for each tumor.

VARIABLE	Value for 103 tumors	>70 Gy (75 tumors)	<70 Gy (68 tumors)	>20 mm (68 tumors)	<20 mm (35 tumors)
INITIAL TUMOR SIZE (T0, mm)^1^	p = 0.001^3^	ns^3^	ns^3^	p = 0.026^3^	ns^2^
Mean	34	27	52	44	15
Range	10-117	10-94	15-117	20-117	10-19
Median	25	22	43	35	15
TUMOR VOLUME (mm)^1^	p = 0.01^2^	ns^3^	ns^3^	p = 0.038^2^	ns^3^
Mean	58	27	142	87	2.5
Range	1-847	1-296	1.6-847	4-847	1-18
Median	9	6	44	22	2
mT/N1 RATIO^1^	p = 0.012^3^	ns^3^	ns^3^	ns^3^	p = 0.001^3^
Mean	2.1	2.2	1.7	1.9	2.3
Range	1.0-6.8	1.1-6.8	1.0-3.2	1.0-5.0	1.1-6.8
Median	1.9	2.0	1.6	1.8	2.1
mT/N2 RATIO^1^	ns^3^	ns^3^	ns^2^	ns^3^	ns^2^
Mean	1.8	1.8	1.8	1.8	1.8
Range	1.0-3.4	1.0-3.4	1.0-3.1	1.0-3.4	1.1-2.8
Median	1.8	1.8	1.7	1.8	1.8
PAD (Gy)^1^	p<0.001^3^	p = 0.005^3^	ns^3^	ns^3^	p = 0.001^3^
Mean	95	110	56	82	120
Range	33-376	71-376	36-69	33-248	61-376
Median	85	94	58	80	103
AAD (Gy)^1^	p<0.001^2^	p = 0.008^2^	ns^2^	p = 0.011^3^	p = 0.028^2^
Mean	84	94	56	76	98
Range	32-168	52-168	34-77	32-168	58-155
Median	78	89	56	71	94
RESPONSE					
CR	7	7	0	2	5
PR	30	26	4	16	14
SD	61	41	20	46	15
PD	5	1	4	4	1

1 - correlations between variable and tumor’s response (according to RECIST1.1)

2 – ANOVA

3 –K-W test

mT/N1 – modified tumor to normal liver ratio in ^99m^Tc-MAA SPECT/CT, mT/N2 – modified tumor to normal liver ratio in ^90^Y-SPECT/CT (bremsstrahlung), PAD - prognostic tumor ^90^Y absorbed dose calculated in 99mTC-MAA SPECT/CT (MIRD), AAD – real tumor ^90^Y absorbed dose calculated in ^90^Y -SPECT/CT (bremsstrahlung)

The overall tumor response rate (ORR_tumor_) was 36% (37 tumors). Their median values of descriptive parameters were as follows: T0 = 19 mm, mT/N1 ratio = 2.1, mT/N2 ratio = 1.8, PAD = 96 Gy, AAD = 89 Gy. The median time to tumor progression (TTP_tumor_) obtained using the Kaplan-Meier estimator was 7 months.

### Standard tumor to normal liver ratio

According to the Kruskal-Wallis test, the ratios sT/N1 (mean 2.87 ± 0.54) and sT/N2 (mean 1.86 ± 0.39) and the derived parameters sPAD (mean 156.74 ± 40.39) and sAAD (mean 109.08 ± 44.46) were all non-significantly associated with tumor response, regardless of its extent (p = 0.053, p = 0.876, p = 0.23, p = 0.077, respectively).

### Modified tumor-to-normal-liver ratio

**Fig 3:**
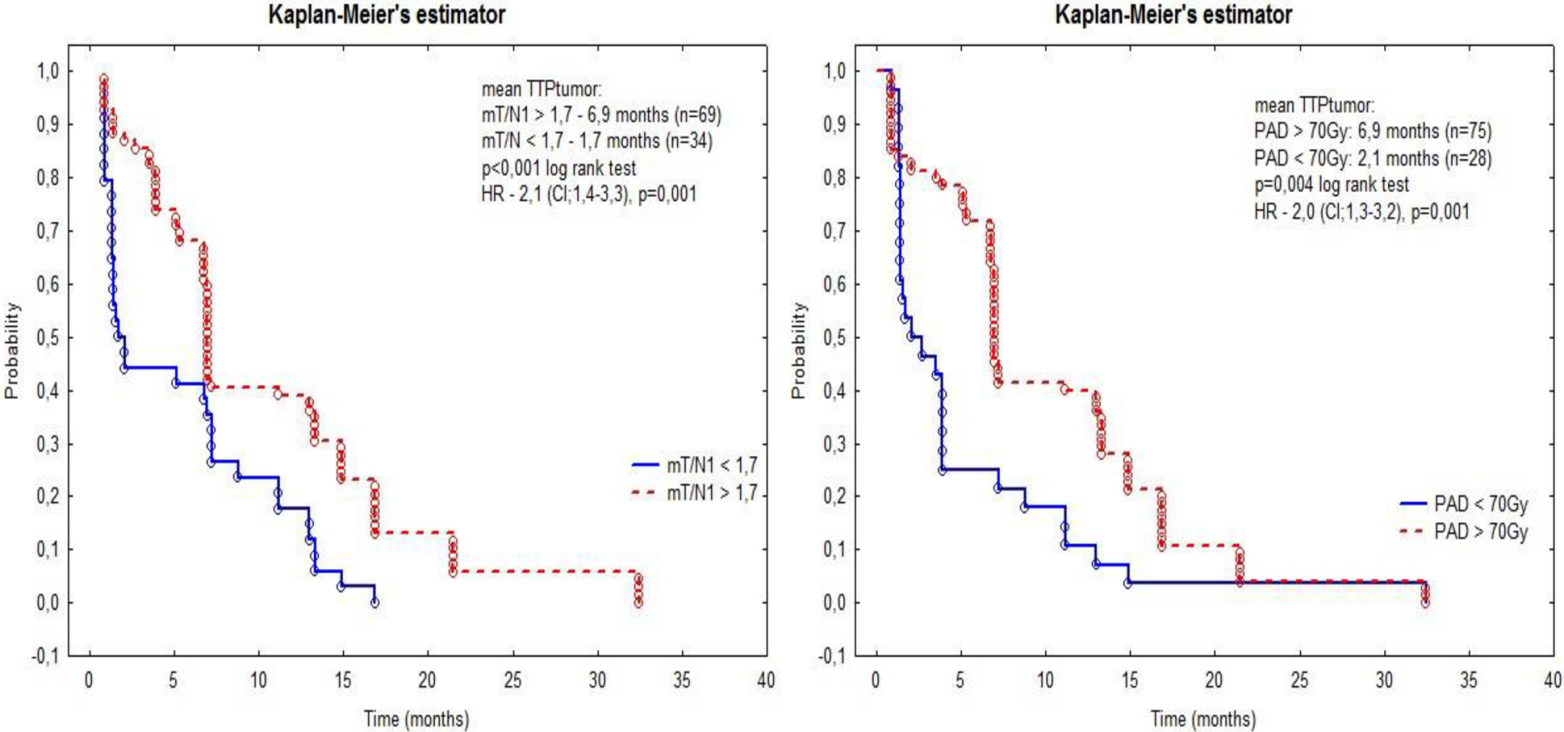
Evaluation of time to tumor progression (TTPtumor) for the threshold of mT/N1 higher or lower than 1.7 (left) and PAD higher or lower than 70Gy (right).

The ratio mT/N1 was higher for tumors that were responsive to treatment (2.4 vs 1.9, p = 0.003) and showed positive correlations with mT/N2 (R: 0.51, p < 0.001), PAD (R: 0.6, p < 0.001), AAD (R: 0.27, p = 0.004) and TTP_tumor_. The ROC curve suggested a cutoff for mT/N1 at the level 1.7 (p = 0.005). TTP_tumor_ was significantly longer for tumors with mT/N1 higher than 1.7 than for tumors with lower ratios (6.9 vs 1.7 months, p < 0.001, log rank test). The risk of progression for tumors with mT/N1 lower than 1.7 was higher than for tumors with higher ratios HR: 2.1, CI:1.4–3.3, p = 0.001) (Fig 3) The ratio mT/N2 was also higher for tumors that were responsive to treatment (1.9 vs 1.8 ns) and showed positive correlations with mT/N1 (R: 0.51, p < 0.001), PAD (R: 0.2, p = 0.038), and AAD (R: 0.43, p < 0.001).

### Predicted and actual tumor ^90^Y absorbed dose

Detailed information on PAD and AAD is presented in Table 4. For 37 tumors that were responsive to treatment, the mean PAD was 113 Gy and the mean AAD was 98 Gy (p < 0.001, Wilcoxon test). For 66 non-responding tumors the means were 82 Gy and 77 Gy, respectively (p<0.05). Negative correlations were found for PAD with T0 (R: - 0.45, p < 0.001), PAD with TUMOR VOLUME (R: - 0.43, p < 0.001), AAD with T0 (R: - 0.41, p < 0.001), and AAD with TUMOR VOLUME (R: - 0.4, p < 0.001). Both PAD and AAD showed significant correlations with tumor response after the SIRT procedure (Kruskal-Wallis test and ANOVA, respectively).

**Fig 4:**
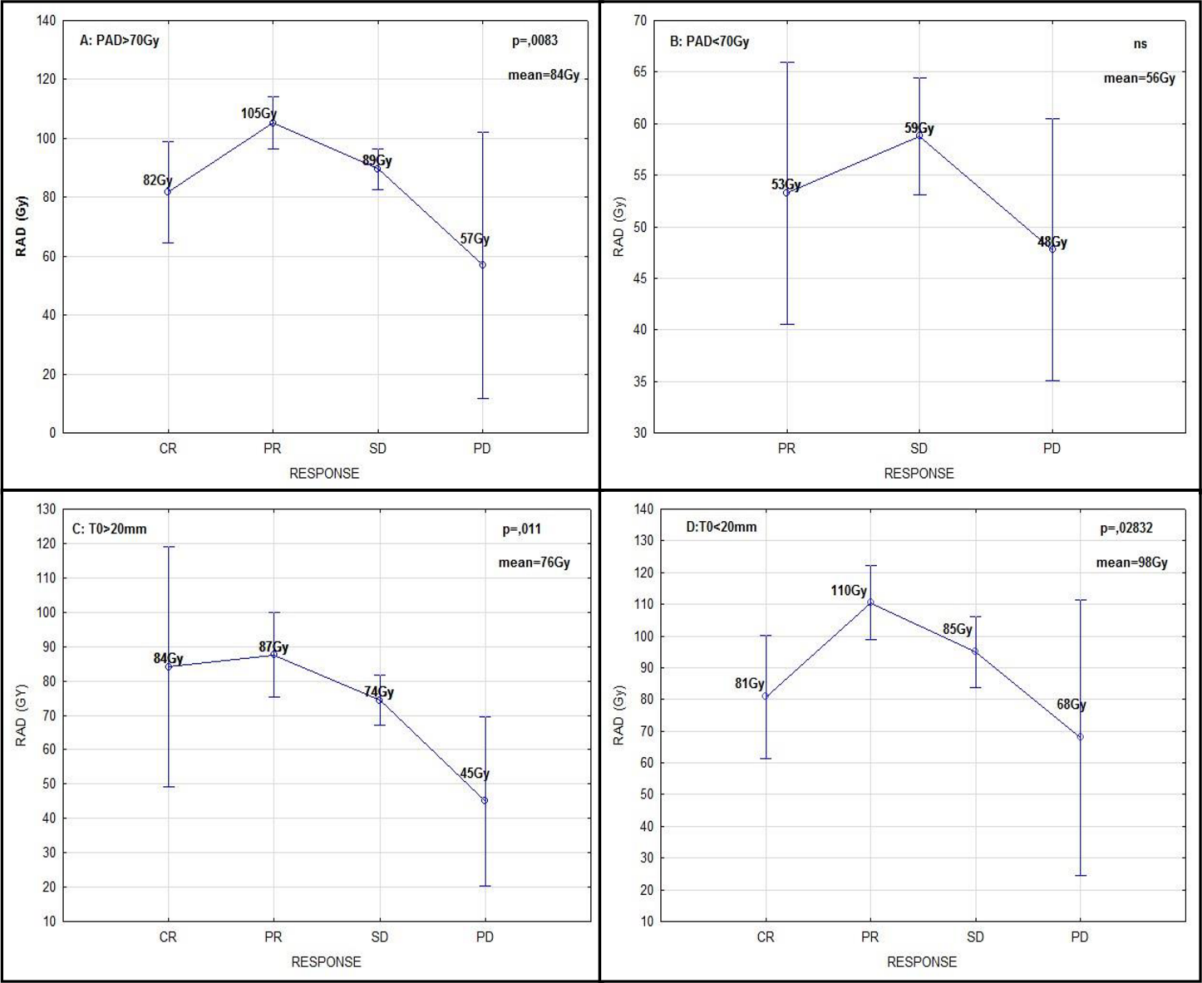
Tumor prognosed absorbed dose (a) and tumor actual absorbed dose (b) distribution depending on tumor response. Tumor prognosed absorbed dose (a) and tumor actual absorbed dose (b) distribution depending on tumor size (threshold at 20mm).

**Fig 5:**
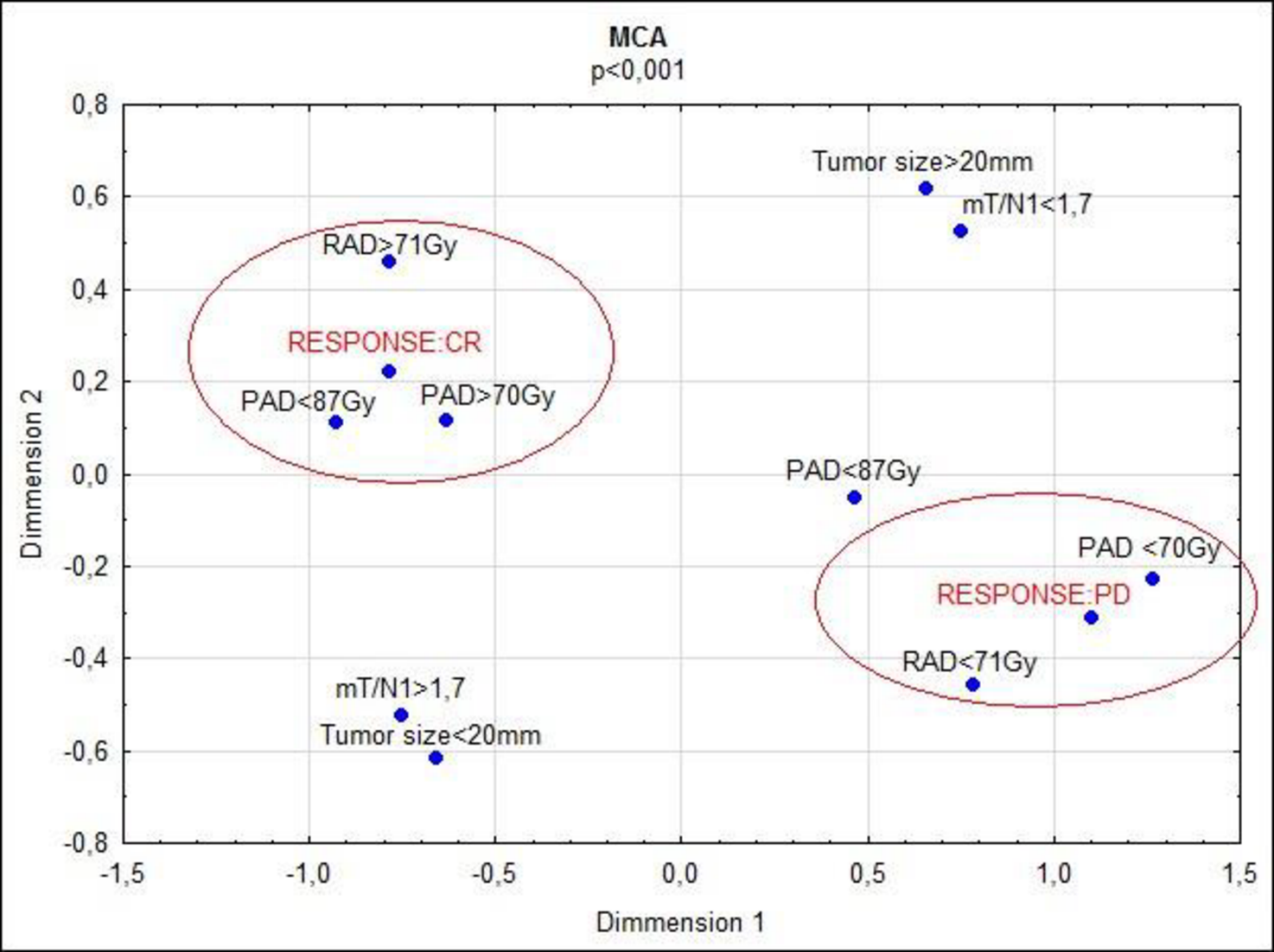
The multivariate correspondence analysis (MCA) for tumors with complete response (CR) and disease progression (PD).

In NIR ANOVA post-hoc tests performed for AAD, significant differences between CR, PR, SD and PD were revealed (e.g., 82 Gy in CR vs 50 Gy in PD, p = 0.031). Results of calculations conducted for tumors with absorbed dose higher or lower than 70 Gy and size smaller or larger than 20 mm are shown in Table 4 and Fig 4. TTP_tumor_ was significantly longer for tumors with PAD higher than 70 Gy (6.9 vs 2.1 months, p = 0.004, log rank test). The risk of progression was elevated for tumors with PAD lower than 70 Gy (HR: 2.0, CI: 1.3–3.2, p = 0.001). The multivariate correspondence analysis performed for tumors with complete response (CR) and progression disease (PD) revealed a large impact of PAD and AAD on these variables. (Fig 5)

## Discussion

The aim of this study was to assess the value of SPECT/CT in predicting the effects of radioembolization for liver tumors, using a simplified method of calculation of the tumor-to-normal-liver ratio (mT/N). SPECT/CT scans are performed two times during the radioembolization procedure. The results showed that if ^99m^Tc-MAA and ^90^Y-microspheres are similar in size, it is possible to predict the actual distribution of the latter in the liver [9, 10].

SPECT/CT imaging can give valuable dosimetric data, crucial for both planning and evaluating SIRT treatment. We have proposed a fast and easy method for dosimetric calculations [13]. The method relies on the RECIST 1.1 criteria, in which the longest tumor diameter (not the tumor volume) is used for further evaluation on follow-up CT scans. These criteria, adopted for SPECT/CT imaging, reflect the notion that counts from the largest lesion diameter accurately reflect the counts in the entire tumor. We confirmed in the SPECT phantom study that gamma counts from the section with the longest tumor diameter are usable to calculate the tumor absorbed dose.

The first step to achieving this goal in the liver is to measure the gamma counts in the ^99m^Tc-MAA and ^90^Y-Bremsstrahlung SPECT/CT slices with the longest tumor diameters and calculate the tumor-to-normal-liver ratios (mT/N1 and mT/N2 respectively). Other dosimetric parameters, such as predicted tumor dose (PAD) and actual tumor dose (AAD), can then be calculated [7, 13–16]. The process of gathering dosimetric parameters is fast, taking only 1-2 minutes for liver tumors. From a dosimetric point of view, the tumor-to-normal-liver ratio is a very interesting parameter because it reflects blood flow in the tumor, as the hepatic artery is the main source of blood supply for liver lesions[1, 17, 18]. This value also forms a basis for calculating further dosimetric parameters, especially PAD for pre-treatment procedures and AAD after radioembolization [7, 14, 16].

As we observed, the mean mT/N1 was 2.1, which means two times greater blood flow in the tumor than in the healthy liver. The mT/N1 ratio was higher for responsive tumors and showed a positive correlation with TTP_tumor_, thus it may be used as a predictor of tumor response. This knowledge should give clinicians an opportunity to plan better treatments, by re-evaluation of the ^90^Y dose before radioembolization. We should remember, however, that calculations made using mT/N1 may sometimes be less accurate due to differences in the distribution of ^99m^Tc-MAA and ^90^Y-microspheres in the liver caused by various factors (e.g., in vivo blood flow variations)[10, 18]. If we take these limitations into account, the use of mT/N1 is simple and gives us the possibility of quick evaluation of conditions of planned ^90^Y treatment. The mT/N2 ratio, which is calculated after radioembolization, would potentially be much more helpful because it reflects the actual ^90^Y dose distribution within liver tumors. Unfortunately, mT/N2 (p = 0.057) is not adequate as a prognostic factor, although its p-value is close to statistical significance and a study with a larger population might give positive results. It is worth mentioning that AAD calculated using mT/N2 showed significant correlation with treatment results.

### Absorbed dose evaluation

Based on mT/N1 and mT/N2 it is possible to calculate PAD and AAD for tumors and liver absorbed dose (LAD) [7, 14]. As we observed in the multiple correspondence analysis, the magnitude of predicted or actual absorbed dose is a major factor determining the tumor response (Fig 5). The mean PAD was 95 Gy, and PAD showed a strong correlation with tumor response. TTP_tumor_ was longer when PAD was higher than 70 Gy. There were 75 tumors with PAD higher than 70 Gy (minimal absorbed dose for tumor destruction). Within this group, there were 7 lesions with CR (all in the entire study) and only one lesion with PD. Interestingly, the median PAD was higher in tumors with PR than with CR. Complete destruction of tumors with lower PAD may be partially explained by a more uniform distribution of microspheres in smaller lesions. Larger, partially necrotic tumors are more resistant, even if they absorb higher ^90^Y doses. The mean AAD was 84 Gy, and AAD was also a predictor of tumor response. Moreover, in terms of AAD all 5 tumors with PD were below the threshold of 70 Gy, whereas for PAD only 4/5 were below this threshold. It makes sense that AAD would better predict tumor response, because this value is calculated after SIRT treatment and reflects the actual ^90^Y-microspheres deposition. We should, however, remember that AAD is determined by the mT/N2 ratio, which was not statistically significant in our study. PAD remains only value calculated before treatment that may predict the response to treatment.

As shown in the Results section, the difference between PAD and AAD in non-responding tumors was not statistically significant. A possible explanation is that non-responders fail to absorb sufficient doses in both the preparatory (^99m^Tc) and treatment phases (^90^Y). In our opinion, the prognostic evaluation of SIRT treatment should be undertaken using all dosimetric parameters, because there is no single prognostic factor that is strongly and reliably predictive of treatment results. Other factors, for example tumor size or volume, may also be important. Consistent with previous reports, our study shows that tumors smaller than 20 mm gather higher mean absorbed doses than larger tumors and show a better response to radioembolization [2, 19, 20].

Information about ^90^Y-microsphere distribution within the liver may be a key to improvement of SIRT treatment results. Using this information, patients prone to potential radiation-induced liver disease (RILD) or patients who accumulate non-curative tumor doses may be identified immediately after the SIRT procedure. Moreover, these data may be useful during treatment planning for much more selective tumor destruction. In other words, mT/N1 and PAD are important, because they allow changes in radioembolization before the procedure itself. Several papers have shown the usefulness of the T/N ratio in prediction of SIRT treatment results. This applies especially in hepatocellular carcinoma, which is known for its developed tumor vasculature and high T/N ratio [21–23]. In comparison to hepatocellular carcinoma, colorectal metastases are characterized by low blood flow and a lower T/N ratio, so there is no easy way of applying dosimetric parameters for assessing tumor response after treatment [18, 24].

As we can observe in previous reports, there are several methods of measuring dosimetric data in SPECT/CT. In the partition model, analysis of ^99m^Tc-MAA distribution between healthy liver and tumor compartments is performed on SPECT images (2D slices reconstructed from 3D projections of the organ), and the activity of ^90^Y is calculated [1, 7, 10, 18, 22, 25]. In some cases, it is difficult to establish proper tumor ROIs (especially in liver with multiple lesions). Moreover, the presence of “hot spots” does not mean there is a tumor in each case; these may be healthy liver areas with higher macroalbumin accumulation. Some authors have used ROIs (or VOIs) with strictly defined size (e.g., 1 cm^2^ or cm^3^, smaller than the tumor size) and defined them on the basis of the researcher’s impressions), while others have administered angiotensin shortly before radioembolization, which should have a significant influence on microsphere deposition in the liver and would considerably alter the pre-treatment procedure [18]. In another study, the authors used only a visual (qualitative) scale of ^90^Y deposition [26]. An interesting new proposal is to use functional dual-tracer SPECT imaging with fusion ^99m^Tc-MAA and ^99m^Tc-Sulphur colloid with semiautomatic tumor segmentation.

A study performed by Lam on 122 patients proved the usefulness of ^99m^Tc-MAA-based dosimetry in prediction of radioembolization results [27]. Within this group, there were 29 patients with metastatic colorectal cancer (which corresponds to 58 tumors investigated based on the RECIST 1.1 criteria). The mean tumor absorbed dose in this study was 30 Gy, although the generally accepted therapeutic dose is much higher, i.e., 70 Gy. This discrepancy may be explained by differences between anatomo-functional and functional methods of gamma count measurement [27, 28]. This implies that there is no simple and direct comparison between these methods. Moreover, the highly sophisticated and time-consuming process of data gathering is in our opinion a barrier to the widespread use of this method.

The above-mentioned methods are based on a standard approach to calculating the tumor-to-normal-liver ratio. The sT/N1 and sT/N2 ratios were proved to be of limited predictive value of tumor response in our study, as they were in majority of previous reports [1, 7, 10, 18, 22, 25]. We have presented a simpler method of dosimetric calculation on a similar cohort of patients (21 patients, 103 tumors). In our opinion, the advantages of gamma count measurement from the scan section with the largest diameter are simplicity, repeatability and representativeness. Our method for calculating the tumor to liver ratio proved to be a valuable tool for predicting treatment results and could serve as a basis for further dosimetric calculations. The resulting information about tumors dosimetric parameters obtained prior to treatment should improve treatment planning, and may help in selecting tumors with insufficient PAD. At this stage, it may be possible to calculate a new slightly higher level of ^90^Y-microsphere activity that may be sufficient to destroy all tumors and still be safe for liver tissue. The alternative approach is to plan much more selective ^90^Y-microsphere injection within tumor areas with PAD lower than 70 Gy to achieve a curative ^90^Y dose. Knowing about insufficient AAD on the day of radioembolization may also allow immediate planning of treatment using other methods (especially radiofrequency ablation), focused only on resistant tumors.

In this study, when we focus on the liver absorbed dose, both predicted and actual doses were almost the same and no serious adverse events were observed. As we can see in the standard guidelines, the limit of liver tolerance is 30 Gy, although some authors have reported that higher doses (up to 70 Gy) were well tolerated by liver tissue [4, 5, 14].

Our study has several limitations. First, we assumed uniform distributions of macroalbumin and microspheres within the liver and tumor compartments. Some tumors had central necrosis areas that did not accumulate the tracer, but these were not excluded from the analysis. Second, we hypothesized that if the method proved to be applicable for varied tumor morphologies, then in a more uniform group, the prognostic value of the results would be greater. Third, even though ROC curve analysis suggested that the cutoff point for PAD should be 87 Gy, we performed calculations using the generally accepted threshold of 70 Gy. Fourth, in accordance with our assumptions regarding the uniformity of dose distribution, gamma counts from the middle (widest diameter) section closely matched those obtained from the entire sphere.

## Conclusions

The mT/N1 ratio and PAD based on ^99m^Tc-MAA SPECT/CT and AAD calculated from ^90^Y-SPECT/CT can be used as predictors of radioembolization results. TTP_tumor_ was significantly longer with mT/N1 greater than 1.7 and for tumors with PAD greater than 70 Gy. The risk of progression was elevated for tumors with mT/N1 lower than 1.7 and PAD lower than 70 Gy. The mT/N2 ratio had no significant correlation with treatment results.

## Supporting information

S1 Table Raw data for the analysis. (XLSX)

## Abbreviations

mT/N1: modified tumor to normal liver ratio in ^99m^Tc-MAA SPECT/CT
mT/N2: modified tumor to normal liver ratio in ^90^Y-SPECT/CT (bremsstrahlung)
PAD: predicted tumor ^90^Y absorbed dose calculated in ^99m^TC-MAA SPECT/CT
AAD: actual tumor ^90^Y absorbed dose calculated in ^90^Y -SPECT/CT (bremsstrahlung)
sT/N1: standard tumor to normal liver ratio in ^99m^Tc-MAA SPECT/CT
sT/N2: standard tumor to normal liver ratio in ^90^Y-SPECT/CT (bremsstrahlung)
sPAD: predicted tumor ^90^Y absorbed dose calculated in ^99m^TC-MAA SPECT/CT based on sT/N1
sAAD: actual tumor ^90^Y absorbed dose calculated in ^90^Y -SPECT/CT (bremsstrahlung) based on sT/N2
CR: complete response (according to RECIST 1.1)
PR: partial response (according to RECIST 1.1)
SD: stable disease (according to RECIST 1.1)
PD: progressive disease (according to RECIST 1.1)
